# Oxidative Carboxylation of 1-Decene to 1,2-Decylene Carbonate

**DOI:** 10.1007/s11244-018-0900-y

**Published:** 2018-01-30

**Authors:** Rebecca V. Engel, Raiedhah Alsaiari, Ewa Nowicka, Samuel Pattisson, Peter J. Miedziak, Simon A. Kondrat, David J. Morgan, Graham J. Hutchings

**Affiliations:** Cardiff Catalysis Institute, School of Chemistry, Cardiff University, Main Building, Park Place, Cardiff, CF10 3AT UK

**Keywords:** Oxidative carboxylation, 1-Decene, 1,2-Decylene carbonate

## Abstract

Cyclic carbonates are valuable chemicals for the chemical industry and thus, their efficient synthesis is essential. Commonly, cyclic carbonates are synthesised in a two-step process involving the epoxidation of an alkene and a subsequent carboxylation to the cyclic carbonate. To couple both steps into a direct oxidative carboxylation reaction would be desired from an economical view point since additional work-up procedures can be avoided. Furthermore, the efficient sequestration of CO_2_, a major greenhouse gas, would also be highly desirable. In this work, the oxidative carboxylation of 1-decene is investigated using supported gold catalysts for the epoxidation step and tetrabutylammonium bromide in combination with zinc bromide for the cycloaddition of carbon dioxide in the second step. The compatibility of the catalysts for both steps is explored and a detailed study of catalyst deactivation using X-ray photoelectron spectroscopy and scanning electron microscopy is reported. Promising selectivity of the 1,2-decylene carbonate is observed using a one-pot two-step approach.

**Electronic supplementary material:**

The online version of this article (10.1007/s11244-018-0900-y) contains supplementary material, which is available to authorized users.

## Introduction

**Scheme 1:**
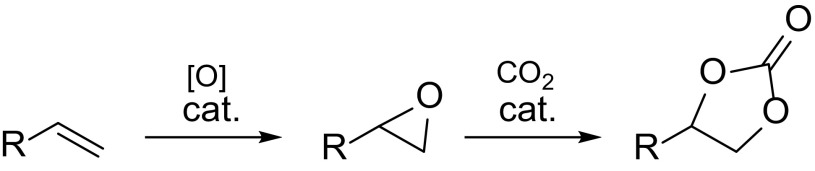
Oxidative carboxylation of alkenes

The increased carbon dioxide concentration in the atmosphere is largely man-made due to the extensive use of fossil fuels like coal, oil, and natural gas. One way towards reduction of carbon dioxide emission to the atmosphere, is the use of concentrated CO_2_ streams produced at industrial locations, as a starting material in chemical synthesis [1] before it is released to the atmosphere. Cyclic carbonates represent a family of compounds where carbon dioxide is used as a feedstock to form value-added chemicals. The application of cyclic carbonates ranges from aprotic polar solvents and electrolytes for lithium-ion batteries to intermediates for pharmaceuticals and raw material for engineering plastics [2–4]. These five-membered rings are usually prepared via the reaction of an epoxide with carbon dioxide, a green C1 building block with 100% atom economy, which can replace hazardous chemicals like phosgene [5]. The synthesis of the epoxides requires alkenes as a starting material. Thus, the direct oxidative carboxylation of olefins (Scheme 1) would be an elegant and economically feasible route to produce cyclic carbonates avoiding work-up procedures in between steps.

The direct synthesis of cyclic carbonates from olefins has been known since 1962 [6]. However, the epoxidation and the cycloaddition of CO_2_ to an epoxide has been mainly studied separately and only a limited amount of literature is available for an integrated approach [3, 7–19]. Most of these studies used *tert*-butyl hydroperoxide (TBHP) [3, 10, 13, 14, 16–18] or hydrogen peroxide [9, 10, 17, 19] as the oxidant. Due to the improved atom efficiency and green nature, molecular oxygen would be the preferred, albeit more challenging, oxidant. In the early 2000’s Aresta et al. published work on the direct oxidative carboxylation of styrene using oxygen as the oxidant applying either homogeneous rhodium complexes or various metal oxides as catalysts [7, 8]. Here, the chosen solvent, dimethylformamide (DMF), was not inert for the epoxidation of alkenes. It was reported that DMF can act as an oxygen transfer agent forming significant amounts of side-products like *N*-formyl-*N*-methylformamide [20, 21] which negates the environmental benefits of using oxygen as the oxidant. Furthermore, Bai et al. described the conversion of different olefins to their corresponding cyclic carbonates using a homogeneous metalloporphyrin catalyst, achieving up to 89% cyclic carbonate yield in the case of styrene [12]. More recently, Kumar et al. reported a cobalt(II) acetylacetonate complex and quaternary triphenylphosphonium bromide catalyst immobilised on magnetic chitosan for the direct oxidative carboxylation of alkenes, using isobutyraldehyde as a sacrificial reductant [15]. The reaction gases, oxygen and carbon dioxide, were introduced sequentially to the reaction mixture and yields up to 85% for both propylene and butylene as starting material were observed.

A major requirement for the integration of the two steps is the compatibility of both the reaction conditions as well as the catalysts for each step. It was previously reported that the tetrabutylammonium bromide catalyst applied for the cycloaddition reaction of CO_2_ to epoxides, deactivates the catalyst used in the epoxidation step, in this case MoO_2_(acac)_2_. This is believed to take place through the bromide-catalysed decomposition of the MoO_2_(acac)_2_ and TBHP which was used as the primary oxidant [13]. To overcome this limitation, the authors added tetrabutylammonium bromide (TBAB) after the completion of the epoxidation reaction.

In the present study, we have investigated the possibility of the integration of two steps of the reaction, namely epoxidation of alkenes and cycloaddition of CO_2_ to epoxides. These studies are performed using highly active supported gold catalysts used for 1-decene epoxidation and the TBAB and zinc bromide catalytic system for the cycloaddition of CO_2_ to the 1,2-epoxydecane, which we expect to be homogeneous under reaction conditions, is investigated.

## Experimental

### Catalyst Preparation

Two grams of 1 wt% supported gold catalysts (denoted 1% Au/support) were prepared via the sol–immobilisation method. Aqueous HAuCl_4_ (4.048 mL, Johnson Matthey, 99.9%; 4.94 mg/mL) were added to deionised water (400 mL) under continuous stirring. Freshly prepared 1 wt% aqueous solution of polyvinyl alcohol (Aldrich, > 99%; PVA/Au = 0.65 (by weight)) was added into the aqueous HAuCl_4_ solution. The mixture was stirred for 15 min, before adding freshly prepared NaBH_4_ solution (2.54 mL, 0.2 M, NaBH_4_/Au = 5 (by mol)) to form a dark-brown sol. The mixture was stirred for an additional 30 min while the pH was adjusted to 2 by dropwise addition of H_2_SO_4_. The support (1.98 g, TiO_2_: P25, Degussa; SiO_2_: Aldrich) was added to the colloidal mixture and stirred for 2 h. The catalyst was filtered, washed thoroughly using approx. 2 L deionised water and then dried at 110 °C for 16 h prior to use.

Graphitic oxide (GO) used in this study was produced via a Hofmann method [22] which was previously optimised for alkene epoxidations by our group [23]. Graphite, (Sigma-Aldrich, < 20 µm, 5 g) was added to a mixture of concentrated sulfuric (75 ml) and nitric acid (25 ml). The slurry was allowed to cool to below 10 °C in an ice bath before stepwise addition of the oxidant. Here, potassium chlorate (30 g) was added over a period of 30 min with vigorous stirring taking care not to exceed 10 °C. After complete addition, stirring was continued for 14 h after which the material was left in air for a further 96 h. For purification of the catalyst, repeated centrifugation and decantation steps were conducted until the solution reached pH 7. The GO was then dried in a vacuum oven for a period of 48 h at 25 °C.

### Catalyst Characterisation

#### X-Ray Photoelectron Spectroscopy (XPS)

X-ray photoelectron spectroscopy was performed on a Kratos Axis Ultra-DLD photoelectron spectrometer using Al(k_α_) radiation, operating at 120 W. Spectra were recorded in the Hybrid spectroscopy mode, using pass energy of 40 eV (0.1 eV step size) for high resolution scans and 80 eV (1.0 eV step size) for survey spectra. Samples were mounted using doubled sided scotch tape attached to glass slides. Charge neutralisation was performed using the Kartos immersion lens system and subsequent calibration of the C (1s) line to 285 eV.

#### Scanning Electron Microscopy (SEM)

Microscopy was performed on a Tescan MAIA 3 field emission gun scanning electron microscope (FEG_SEM) fitted with secondary and backscattered electron detection. Energy-dispersive X-ray (EDX) analysis and mapping was done using Oxford Instruments X-Max^N^ 80 detector and the data analysed using the Aztec software. Samples were mounted on silicon wafers and affixed to aluminium stubs using adhesive carbon tape. The samples were analysed uncoated.

### Reaction Procedures

Epoxidation reactions were carried out either in a magnetically stirred, roundbottomed glass flask reactor (50 mL capacity) fitted with a reflux condenser or a 50 mL Parr stainless steel autoclave equipped with a Teflon inlet. 1-decene (Sigma Aldrich, ≥ 94%; 10 mL, 53 mmol) was added to the reactor together with the catalyst (0.1 g) and AIBN (Aldrich, 97.5%; 0.006 g, 0.036 mmol). In the autoclave reactions, the autoclave was purged three times with nitrogen before being pressurised with 15 bar oxygen. The reaction mixture was heated to 90 °C under continuous stirring for 24 h. After being cooled to room temperature, the solution was filtered and products were analysed by gas chromatography using a Varian star 3800 CX gas chromatograph with a CP-wax 52 column (capillary column, 25 m, 0.35 mm ID, 0.2 micron) coupled with a flame ionisation detector (FID). The reactions using the GO catalyst were conducted without a radical initiator for a period of 48 h. Inductively coupled plasma mass spectrometry (ICP-MS) was performed on an Agilent 7900 ICP-MS equipped with a micromist nebuliser in organic phase mode. Quantification was carried out by comparison with a calibration curve.

The cycloaddition reaction was performed in a 50 mL Parr stainless steel autoclave equipped with a Teflon inlet. 1,2-epoxydecane (Aldrich, 98%; 5 mL, 27 mmol), TBAB (Alfa Aesar, ≥ 98%; 0.18 g, 0.56 mmol) and zinc bromide (Alfa Aesar, 98%; 0.08 g, 0.335 mmol) were added into the autoclave. The reactor was purged three times with CO_2_ before being charged with 20 bar CO_2_. Then the reaction mixture was heated to 80 °C under continuous stirring for 4 h. After being cooled to room temperature, the solution was filtered and quantitative analysis of the reaction mixture was performed using gas chromatography (details as mentioned above). For qualitative analysis, gas chromatography coupled with mass spectrometry (Walters GCT Premier GC coupled with a HP 6890N MS) was used.

The one-pot synthesis of cyclic carbonate was performed in a 50 mL Parr stainless steel autoclave equipped with a Teflon inlet. The reactor was charged with 1-decene (10 mL, 53 mmol), 1% Au/support (0.1 g), AIBN (0.006 g, 0.04 mmol), TBAB (0.36 g, 1.12 mmol), and zinc bromide (0.16 g, 0.71 mmol). The autoclave was purged three times with oxygen before being charged with 15 bar oxygen and 15 bar CO_2_ and heated to 90 °C for 28 h. After being cooled to room temperature, the solution was filtered and products analysed by gas chromatography as reported before.

In the one-pot two-step protocol, first the epoxidation reaction was carried out either in a round bottom flask or autoclave as described above. After 24 h reaction, the reactor was cooled to room temperature and depressurised. Then the catalysts TBAB (0.36 g, 1.12 mmol) and zinc bromide (0.16 g, 0.71 mmol) were added to the reaction vessel. After closing the reactor it was pressurized with 20 bar CO_2_, it was heated to 90 °C under continuous stirring for a period of 4 h. After being cooled to room temperature, the solution was filtered and products were analysed by gas chromatography.

The experiments have been repeated up to three times and the standard deviation was up to 2%. The mass balance for the experiments was calculated to be over 80% in all cases. The mass loss is due to cracking of 1-decene leading to gaseous products (light hydrocarbons and CO_x_) as reported previously [24] as well as because of the averaged response factor assigned to the small amount of unidentified products. For the GC analysis of the product solution, mesitylene was used as standard and all known compounds were calibrated. Conversions (X) were determined using corrected GC counts by the following equation:

1 $$X=\left( {1 - \frac{{\sum {Substrate} }}{{\left( {\sum {Products+Substrate} } \right)}}~} \right) \times 100\%$$

and selectivities (S) were calculated based on corrected GC counts of observed products using Eq. (2).

2 $$S=\frac{{Product}}{{\sum {Products} }} \times 100\%$$

## Results and Discussion

### Direct Oxidative Carboxylation: One-Pot Approach

In this study, 1% Au/SiO_2_ and Au/TiO_2_ catalysts, prepared by sol-immobilisation, have been tested in the solvent-free epoxidation of 1-decene using AIBN as a radical initiator with atmospheric pressure of air as the oxidant (Table 1, entry 2 and 3). In comparison to the blank reaction where only the radical initiator is present (Table 1, entry 1), an increase in both the conversion and epoxide selectivity is observed. The increase of conversion from 5 to 13% and selectivity from 17 to 34%, can be assigned to the catalytic activity of the supported gold catalysts. This level of catalytic performance observed in oxide supported gold is comparable to the previously reported graphite supported gold material yielding of 31% epoxide selectivity at 12% conversion [24]. Observed side products are the diol, allylic compounds (1-decen-3-one, 1-decen-3-ol, 2-decenal, 2-decen-1-ol), and other C_7_–C_10_ products such as 1-heptanol, ocatanoic acid, 3-nonene-1-ol, or 2-decenoic acid. Sun et al. previously reported excellent results in the direct oxidative carboxylation of styrene, a similar approach could be used in the direct carboxylation of 1-decene. In the work reported by the group, yields of styrene carbonate up to 42% could be obtained in a one-pot approach using a Au/SiO_2_-ZnBr_2_/Bu_4_NBr catalyst system and TBHP as the primary oxidant [3]. Due to the good catalytic performance of the heterogeneous 1% Au/SiO_2_ catalyst in the aerobic epoxidation of 1-decene, a one-pot approach of 1, 2-decylene carbonate synthesis in the presence of TBAB and zinc bromide, which are expected to be homogeneous under reaction conditions, was attempted, keeping oxygen as the oxidant (Table 1, entry 4). Unfortunately, a lower than expected selectivity of 8% to the 1,2,-decylene carbonate was observed, based on the stoichiometric conversion of the epoxide. Therefore, the individual steps of the overall reaction were investigated using the combination of the catalysts from both steps to identify any deactivation issues.

**Table 1:** 1-decene epoxidation and direct oxidative carboxylation using 1% Au/support and 1% Au/SiO_2_-Bu_4_NBr-ZnBr_2_, respectively

Entry	Reaction	${{\text{p}}_{{{\text{O}}_2}}}$ (bar)	${{\text{p}}_{{\text{C}}{{\text{O}}_2}}}$ (bar)	Conversion (%)	Selectivity (%)	
					Epoxide	Cyclic carbonate
1	Blank	–	–	5	17	–
2	1% Au/SiO_2_	–	–	13	34	–
3	1% Au/TiO_2_	–	–	13	34	–
4^a^	1% Au/SiO_2_-ZnBr_2_/Bu_4_NBr	15	15	20	3	8

Reaction conditions: 1% Au/SiO_2_ (0.1 g), 1-decene (10 mL, 53 mmol), AIBN (6 mg, 0.036 mmol), 800 rpm, 90 °C, 24 h

^a^Bu_4_NBr (0.36 g, 1.12 mmol) and ZnBr_2_ (0.16 g, 0.71 mmol) added directly at the beginning of the reaction; 28 h

### Cycloaddition of CO_2_ and 1,2-Epoxydecane in the Presence of Supported Gold Catalysts

The supported gold catalysts have been applied in the cycloaddition of CO_2_ to 1,2-epoxydecane to study their influence on cyclic carbonate formation. Neither of the epoxidation catalysts, Au/TiO_2_ nor Au/SiO_2_, were active in the synthesis of cyclic carbonate under the present conditions (Table 2, entry 2 and 3), despite being used in higher quantities (0.2 g) than were used for the epoxidation reaction reported previously (0.1 g). The carboxylation catalytic system of TBAB and zinc bromide lead to 98% cyclic carbonate selectivity at almost full conversion (Table 2, entry 4). This excellent result wasn’t affected by the addition of the supported gold catalyst (Table 2, entry 5 and 6). The 1% difference in cyclic carbonate selectivity in the presence of Au/TiO_2_ is well within the error range of the analysis method. Thus, the supported gold catalyst didn’t influence the activity of the TBAB and zinc bromide catalytic system, which was a good indicator for further optimization of the reaction.

**Table 2:** Effect of supported gold catalysts on the cycloaddition of carbon dioxide to 1,2-epoxydecane

Entry	Catalyst	Conversion (%)	Cyclic carbonate selectivity (%)
1	Blank	0	0
2	1% Au/TiO_2_^a^	1	0
3	1% Au/SiO_2_^a^	1	0
4	Bu_4_NBr + ZnBr_2_	98	98
5	1% Au/SiO_2_ + Bu_4_NBr + ZnBr_2_	98	98
6	1% Au/TiO_2_ + Bu_4_NBr + ZnBr_2_	98	97

Reaction conditions: supported gold catalyst (0.2 g), 1,2-epoxydecane (5 mL, 26.88 mmol), Bu_4_NBr (0.18 g, 0.56 mmol), ZnBr_2_ (0.08 g, 0.335 mmol), 20 bar CO_2_, 80°C,4 h, 800 rpm

^a^90 °C

### Epoxidation of 1-Decene in Presence of TBAB and Zinc Bromide

Since the formation of cyclic carbonates was not influenced by the supported gold catalyst the epoxidation step has been investigated in more detail with TBAB and zinc bromide present in the reactor. The synthesis of cyclic carbonates from epoxides are usually performed under high CO_2_ pressure in an autoclave. Thus, the one-pot reaction described above has been performed using overpressure. When the epoxidation of 1-decene is performed using 15 bar oxygen the conversion increases but the epoxide selectivity decreases to 19% (Table 3, entry 2) in comparison to the 34% observed for the epoxidation reaction at ambient pressure. Additionally, the comparison of the catalysed reaction with the blank reaction (Table 3, entry 1) under these conditions shows that there is no distinct increase when the supported gold catalyst is applied. The reason for this is most likely that epoxidation by the catalyst is masked by the allylic oxidation of the uncatalysed reaction. Furthermore, at the elevated oxygen pressure the amount of cracked products (summarised under others in Table 3) increased, which leads to lower epoxide selectivity. When TBAB and zinc bromide are added to the epoxidation reaction, the epoxide selectivity decreases further and the selectivity of the diol and the other products increases. In this case only 2% epoxide selectivity is observed after 24 h reaction time.

**Table 3:** Effect of TBAB and zinc bromide on 1-decene epoxidation using gold catalysts at 15 bar O_2_

Entry	Catalyst	Co-catalyst	Conversion (%)	Selectivity (%)			
				Epoxide	Allylic products	Diol	Others
1	Blank	–	22	17	28	0	40
2	1% Au/SiO_2_	–	24	19	24	2	41
3	1% Au/SiO_2_	Bu_4_NBr + ZnBr_2_	21	2	26	7	55

Reaction conditions: 1% Au/SiO_2_ (0.1 g), 1-decene (10 mL, 53 mmol), AIBN (6 mg, 0.036 mmol), Bu_4_NBr (0.36 g, 1.12 mmol), ZnBr_2_ (0.16 g, 0.71 mmol), 90 °C, 15 bar O_2_, 24 h, 800 rpm. Allylic products = ∑(1-decen-3-one, 1-decen-3-ol, 2-decenal, 2-decen-1-ol). Others = ∑ of all identified products including (C_7_ + C_8_ + C_9_ acids, C_8_ + C_9_ aldehyde, C_7_ + C_8_ alcohols, 3-nonen-1-ol, 3-nonanone, cyclododecane, 2-decenoic acid, epoxide ring opening products such as bromoalcohol)

As the results for the epoxidation reaction are more promising when it is performed under ambient pressure, the effect of TBAB and zinc bromide have also been studied under these conditions. When the quaternary ammonium salt and zinc bromide are added to a blank epoxidation reaction, where only the radical initiator (AIBN) is present, the conversion decreases and no selectivity towards the epoxide is observed, instead mainly allylic products are formed (Table 4, entry 2). Unfortunately, the addition of the salts also deactivate the supported gold catalysts (Table 4, entry 4 and 6). In particular, the epoxide selectivity decreases to 9 and 12% for the Au/SiO_2_ or Au/TiO_2_ catalyst, respectively. At the same time the selectivity to the diol and other products including the bromoalcohol, due to ring opening by bromide, increase.

**Table 4:** Effect of TBAB and zinc bromide on 1-decene epoxidation using supported gold catalysts under atmospheric pressure of air

Entry	Catalyst	Co-catalyst	Conversion (%)	Selectivity (%)			
				Epoxide	Allylic products	Diol	Others
1	Blank	–	5	17	45	1	25
2	Blank	Bu_4_NBr + ZnBr_2_	1	0	71	0	29
3	1% Au/SiO_2_	–	13	34	28	1	27
4	1% Au/SiO_2_	Bu_4_NBr + ZnBr_2_	10	9	32	7	39
5	1% Au/TiO_2_	–	13	34	30	2	24
6	1% Au/TiO_2_	Bu_4_NBr + ZnBr_2_	10	12	32	6	44

Reaction conditions: supported gold catalyst (0.1 g), 1-decene (10 mL, 53 mmol), AIBN (6 mg, 0.036 mmol), Bu_4_NBr (0.36 g, 1.12 mmol), ZnBr_2_ (0.16 g, 0.71 mmol), 90 °C, atmospheric pressure of air, 24 h, 800 rpm. Allylic products = ∑ (1-decen-3-one, 1-decen-3-ol, 2-decenal, 2-decen-1-ol). Others = ∑ of all identified products including (C_7_ + C_8_ + C_9_ acids, C_8_ + C_9_ aldehyde, C_7_ + C_8_ alcohols, 3-nonen-1-ol, 3-nonanone, cyclododecane, 2-decenoic acid, epoxide ring opening products such as bromoalcohol)

Based on the data reported in Table 4, the presence of the catalysts used for the cycloaddition reaction leads to a decrease in the selectivity in the epoxidation reaction. Therefore, it is clear, that the TBAB and zinc bromide must act as a poison for Au materials, which suggests that a one pot approach in the synthesis of carbonates from alkenes, may not be achieved using the current catalytic system. Thus, the nature of deactivation of Au catalysts was investigated in order to understand its cause and to determine whether it can be further prevented. The visual observation of the reaction conducted in the round-bottom flask was found to be helpful to understand the deactivation phenomenon; when the salts are added to the 1-decene reaction solution, a rapid agglomeration and immobilisation of the catalyst onto the flask walls occurs within 5 min of reaction. Furthermore, during the course of the reaction the Au/TiO_2_ catalyst, which has a light purple/grey colour, loses its shade and becomes white which may indicate leaching of the gold. However, ICP-MS analysis revealed less than 1 ppm gold in the product solution accounting for only less than 1% leaching of the total gold content of the Au/TiO_2_ catalyst. Thus, the colour change might me due to deposition of the white salts onto the surface of the catalyst deactivating the active sites. As GO catalysts have been reported to be active in the epoxidation of 1-decene without the need of supported gold or radical initiator [23], we decided to investigate a GO catalyst prepared by the Hofmann method in the presence of TBAB and zinc bromide (Table 5, entry 4). As with the supported gold catalyst, the GO is deactivating during the reaction leading to a decrease in conversion from 12 to 2% and epoxide selectivity from 24 to 0%. Additionally, the influence of zinc bromide and TBAB have been investigated individually (Table 5, entry 2 and 3). In both cases the conversion is significantly reduced, however, a difference in product distribution can be observed when the catalyst are used separately. While with TBAB mainly allylic compounds are formed; the diol, nonanoic acid, 3-nonene-1-ol, octanoic acid, and heptanoic acid are the main products formed in the presence of ZnBr_2_. When the amount of co-catalyst added to the reaction, was reduced by a factor of 10 (Table 5, entry 5) the conversion of 1-decene was hardly affected. However, the epoxide selectivity reduced significantly from 24 to 16%, suggesting that even the lower amounts of TBAB could be inhibiting the ability of the GO catalyst to direct the breakdown of the hydroperoxide intermediate towards the epoxide and promoting the dehydration of the intermediate to allylic products. In fact, it is known that quaternary ammonium salts can decompose hydroperoxides [25]. ZnBr_2_ is likely promoting the over-oxidation of the epoxide to the diol and cracked products. This is most likely also the reason for the reduced epoxide selectivity when the supported gold catalysts are applied together with the catalysts for the second step (Table 4).

**Table 5:** Effect of TBAB and zinc bromide on 1-decene epoxidation using GO as catalyst under atmospheric pressure of air

Entry	Catalyst	Co-catalyst	Conversion (%)	Selectivity (%)			
				Epoxide	Allylic products	Diol	Others
1	GO	–	12	24	31	4	28
2	GO	ZnBr_2_	2	0	38	3	50
3	GO	Bu_4_NBr	1	0	96	0	0
4	GO	Bu_4_NBr + ZnBr_2_	2	0	53	4	42
5	GO	Bu_4_NBr + ZnBr_2_^a^	11	16	34	5	30

Reaction conditions: GO (0.1 g), 1-decene (10 mL, 53 mmol), Bu_4_NBr (0.18 g, 0.56 mmol), ZnBr_2_ (0.08 g, 0.36 mmol), 90 °C, atmospheric pressure of air, 48 h, 800 rpm. Allylic products = ∑ (1-decen-3-one, 1-decen-3-ol, 2-decenal, 2-decen-1-ol). Others = ∑ of all identified products including (C_7_ + C_8_ + C_9_ acids, C_8_ + C_9_ aldehyde, C_7_ + C_8_ alcohols, 3-nonen-1-ol, 3-nonanone, cyclododecane, 2-decenoic acid, epoxide ring opening products such as bromoalcohol)

^a^Bu_4_NBr (0.018 g, 0.056 mmol), ZnBr_2_ (0.008 g, 0.036 mmol)

As described earlier with the supported gold catalysts, the GO agglomerates and sticks to the walls of the glass reactor shortly after the start of the reaction, when both salts are present. The addition of TBAB leads to the same GO behaviour, while the agglomeration is less pronounced and takes much longer with the presence of zinc bromide in the reaction mixture. A potential explanation for this could be the formation of micellar type structures in the presence of TBAB. In the non-polar environment of 1-decene, the polar salts stick to the surface of the polar solid catalyst and the hydrophobic butyl groups of TBAB reach into the 1-decene solution forming a micelle like structure, encapsulating the catalyst. Again, the addition of significantly less TBAB and zinc bromide, does not influence the reaction as much and the catalyst does not noticeably agglomerate over the 48 h.

**Fig. 1:**
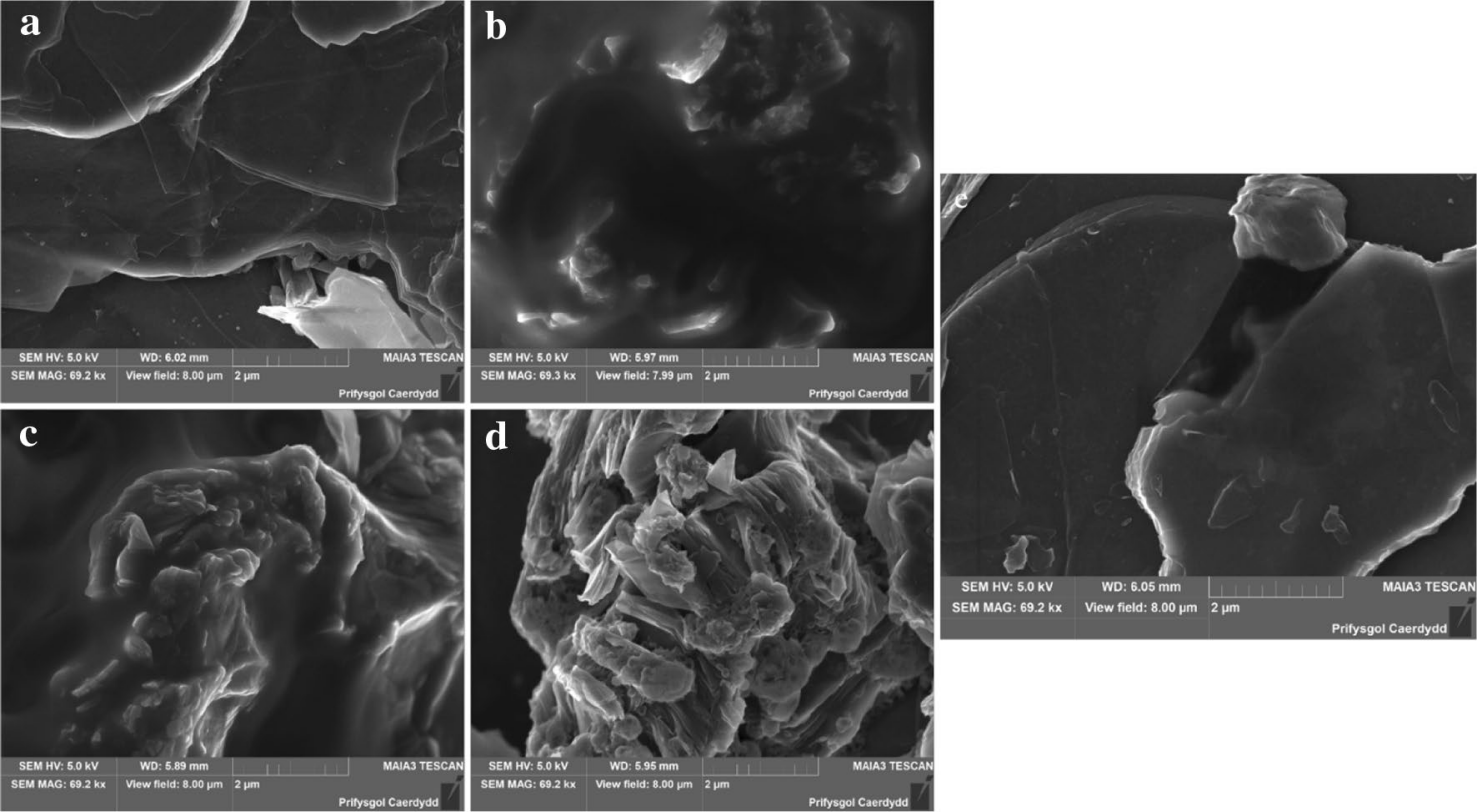
SEM secondary electron micrographs of the fresh (**a**) and spent GO catalysts (**b** Bu_4_NBr + ZnBr_2_, **c** Bu_4_NBr, **d** ZnBr_2_, **e** 10× less Bu_4_NBr + ZnBr_2_)

SEM and XPS analysis have been performed on the fresh and the spent catalysts in order to better understand the deactivation mechanism. Figure 1 displays SEM images taken of the GO catalyst after being used in the epoxidation reaction together with TBAB and/or zinc bromide. As can be seen in Fig. 1a, the fresh catalyst has a layered plate-like structure which is preserved when only small amounts of TBAB and zinc bromide are present. The morphology changes significantly when standard amounts of the salts are added to the reaction mixture. An organic, almost polymer-like, layer covers the typical GO structure when TBAB is present as shown in Fig. 1b, c. When only zinc bromide is added to the reaction mixture the surface is not covered by a smooth layer, but crystal-like growths can be observed on the surface (Fig. 1d). These layers and changes in morphology could be the reason for the low activity in the epoxidation reaction by blocking the catalyst surface.

**Fig. 2:**
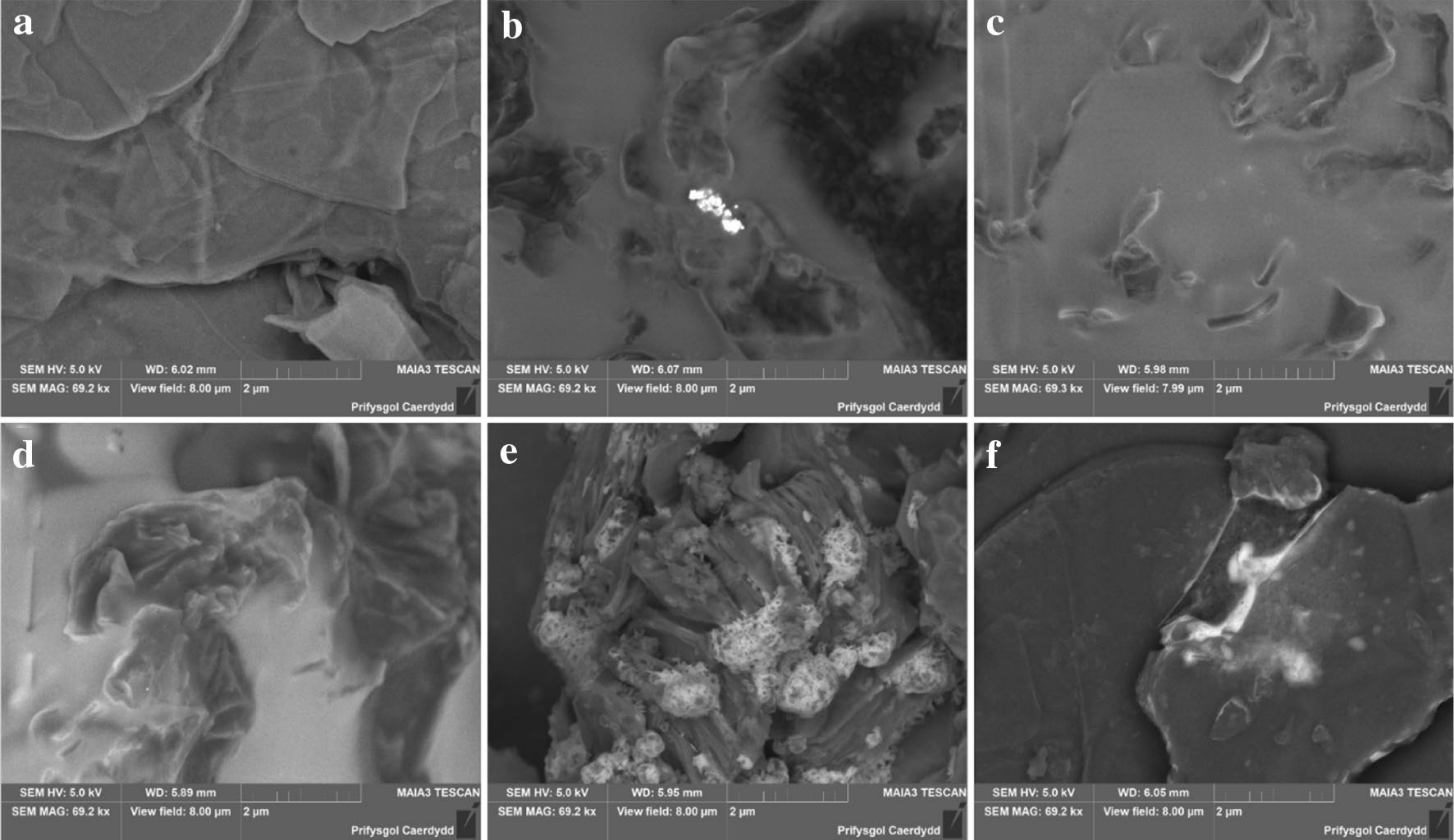
SEM backscattered electron micrographs of the fresh **a** and spent catalysts (**b** and **c** Bu_4_NBr + ZnBr_2_, **d** Bu_4_NBr, **e** ZnBr_2_, **f** 10× less Bu_4_NBr + ZnBr_2_)

The use of a back scattered electron detector (BSD) for SEM imaging can shed light on the distribution of the heavier elements zinc and bromine (Fig. 2). The light colour of the layer covering most of the GO catalyst after use with TBAB and zinc bromide (Fig. 2b, c) suggests a quite uniform distribution of bromine and zinc. However, some lighter spots where most likely only zinc is accumulated are also observed. This is similar for the sample with no zinc present (Fig. 2d), where the light grey colour suggests an even distribution of bromine in the covering layer. The darker areas displayed on the picture, represent surface GO fragments. The crystal-like growth observed in the sample where zinc bromide only was present during the epoxidation is also rich in heavier elements, most likely zinc and bromine (Fig. 2e). In Fig. 2f light areas indicate an accumulation of zinc and bromine on the GO surface for the sample exposed to the lower quantities of TBAB and zinc bromide as well.

These findings are underlined by EDX mapping (Figs. S1–S4 in the supporting information). There is an even distribution of zinc and bromine in the spent GO sample used together with TBAB and zinc bromide (Fig. S1) while the high oxygen content correlates with the higher carbon content coinciding with darker areas in the SEM image using a BSD representing GO fragments emerging from the carbonaceous layer. A similar finding for the GO sample used with TBAB addition only can be seen in Fig. S2. Interestingly, in the sample of the spent GO catalyst used with only zinc bromide added to the reaction mixture, the oxygen rich areas coincide with the zinc rich areas as well as the light areas in SEM image (Fig. S3). Thus, the formation of ZnO on the surface of the GO catalyst is likely and could explain the difference in morphology but also immobilisation of ZnBr_2_ at surface oxygen species of the GO catalyst are possible.

XPS supports the conclusions from the SEM analysis. The carbon binding energies for the typical GO catalyst representing the carbon–carbon bonding (285 eV) as well as hydroxyl functionalities on the surface (287 eV) change for all used samples (Fig. S5 in the supporting information). The peak shape alter significantly from the fresh GO catalyst but also new peaks at higher binding energies can be observed indicating multiple C–O functionalities suggesting a more polymer-like surface, this is concurrent with varying oxygen content for each sample. The Zn(2p_3/2_) binding energies for the samples with low TBAB, zinc bromide and zinc only addition to the reaction is 1022.8, 1 eV higher than the sample with only the standard amount of TBAB and zinc bromide added to the epoxidation (1021.8 eV). The higher binding energy we assign to the formation of ZnBr_x_ and consistent with binding energies observed for zinc bromide [26] and supported by multiple Br states noted in the Br(3d) spectra. The lower binding energy Zn species is typical of ZnO [27] or bromozincate type species [28] *via* complexation with the surface. This is contradictory to the SEM EDX analysis in which especially the zinc bromide only addition samples suggest ZnO formation. Yet, XPS is a surface sensitive technique while the SEM analysis of the carbon material under these conditions is more a bulk analysis. Thus, it is possible that there is zinc bromide on the surface while there is zinc oxide closer to the bulk; again this is supported by the presence of multiple Br states, which we assign to Zn–Br and C–Br bonds, for all samples but for that in the used GO sample with the standard amounts of TBAB and zinc bromide added to the reaction solution, where a single bromine state is found consistent with that of C–Br (ca. 68 eV).

Considering these findings on the deactivation of the GO catalyst the difference in product selectivity could be explained. The polymer-like layer covering the GO catalyst when TBAB is present in the reaction solution just deactivates the catalyst and the low amounts of 1-decene are mainly converted to the allylic products due to hydroperoxide decomposition by TBAB (Table 3, entry 3) as mentioned above. However, with zinc bromide only addition, either the formed ZnO or immobilised ZnBr_2_ on the surface could be the reason for the increased selectivity to cracking products, such as nonanoic acid and 3-nonene-1-ol and over-oxidation to the diol (Table 3, entry 2).

### Direct Oxidative Carboxylation of 1-Decene: One-Pot Two-Step Approach

The prevention of the deactivation of the solid catalysts for the epoxidation reaction with TBAB and zinc bromide in the system, remains a challenge. Thus, a one-pot two-step approach, similar as reported by Chen et al. [13], has been investigated for the direct oxidative carboxylation of 1-decene avoiding the deactivation of the supported gold catalyst by the TBAB and zinc bromide through sequential addition. Unfortunately, when the epoxidation reaction was performed under elevated oxygen pressure, the cyclic carbonate selectivity was low despite the fact that the TBAB and zinc bromide weren’t present for the first 24 h. A reason could be the formation of by-products which either deactivate the TBAB and zinc bromide catalysts or facilitate the ring opening of the epoxide. However, when the epoxidation step is first performed under atmospheric pressure, the cyclic carbonate selectivity reaches 24% as shown in Table 6.

**Table 6:** One-pot two-step approach for the direct synthesis of cyclic carbonate starting from 1-decene using 1% Au/support-Bu_4_NBr-ZnBr_2_

Entry	${{\text{p}}_{{{\text{O}}_2}}}$ (bar)	${{\text{p}}_{{\text{C}}{{\text{O}}_2}}}$ (bar)	Conversion (%)	Selectivity (%)	
				Epoxide	Cyclic carbonate
1	15	20	24	2 (19)^a^	9
2	–^b^	20	14	3 (34)^a^	24
3^c^	–^b^	20	14	3 (34)^a^	22

Reaction conditions: 1% Au/SiO_2_ (0.1 g), Bu_4_NBr (0.36 g, 1.12 mmol), ZnBr_2_ (0.16 g, 0.71 mmol), 1-decene (10 mL, 53 mmol), AIBN (6 mg, 0.036 mmol), 800 rpm, 90 °C, 24 h epoxidation + 4 h cycloaddition of CO_2_ with the formed 1,2-epoxydecane. Catalyst for the cycloaddition was added after the epoxidation step

^a^Epoxide selectivity after epoxidation step in brackets

^b^Atmospheric pressure of air. Reaction solution was transferred into an autoclave after the epoxidation

^c^1% Au/TiO_2_ (0.1 g)

The superior cyclic carbonate selectivity observed by Sun et al. [3] using a similar catalytic system can be rationalised by a number of factors. The most important difference is that of the inherent reactivities and structural differences between styrene and 1-decene. The absence of an allylic hydrogen in styrene allows for a greater selectivity to epoxide to be achieved than in 1-decene. The presence of an allylic hydrogen in 1-decene and use of oxygen ultimately limits carbonate yield to 50% due to the equimolar formation of epoxide and allylic alcohol from the hydroperoxide intermediate. The use of TBHP as an oxidant will of course be far more selective for the direct epoxidation of the alkene. Sun et al. also discussed the importance of choice of oxidant, stating that TBHP led to greater yields of carbonate over H_2_O_2_ [10]. This may also hint towards the sensitivity of these systems towards water. The presence of in-situ formed water formed via breakdown of decenyl hydroperoxide would likely lead to hydration of the epoxide to the diol rather than formation of cyclic carbonate. The deactivation of epoxidation catalysts observed in this study may be avoided by use of ionic liquids as used by Sun et al. [10] or other solvent systems, however this would decrease the green nature and scalability of the overall process. For the oxidative carboxylation of 1-decene, a two-step protocol seems to be the best approach at the present time. However, given that the deactivation of the GO catalyst was much less pronounced when significantly less TBAB and zinc bromide were present, this might be another route to follow for enhanced 1,2-decylene carbonate selectivity in a one-pot approach.

## Conclusion

In the current work active gold catalysts supported on SiO_2_ and TiO_2_ were reported for the epoxidation of 1-decene. A one-pot approach for the oxidative carboxylation of 1-decene using TBAB and zinc bromide in addition to the supported gold catalyst resulted in low 1,2-decylene carbonate selectivity due to deactivation of the catalyst. While the cycloaddition step is not influenced by the gold catalyst, the epoxide selectivity significantly decreases when TBAB and zinc bromide are present. A detailed deactivation study revealed the formation of an organic polymer-like layer on the surface of the catalyst when TBAB is present, and a change in morphology when only zinc bromide is added to the reaction mixture. These deactivation issues can be avoided by using a one-pot two-step approach for the oxidative carboxylation of 1-decene. The cyclic carbonate selectivity can particularly be enhanced when the epoxidation step is performed under atmospheric pressure of air.

## Electronic Supplementary Material

Below is the link to the electronic supplementary material.

Supplementary material 1 (DOCX 3178 KB)
