# Supplementary material for: Oxidative Carboxylation of 1-Decene to 1,2-Decylene Carbonate
Source: Top Catal. 2018 Jan 30;61(5):509–18. doi: 10.1007/s11244-018-0900-y (PMC6560682; doi:10.1007/s11244-018-0900-y)
Supplement: Supplementary file 1 — Supplementary material 1 (DOCX 3178 KB) [file 11244_2018_900_MOESM1_ESM.docx]

**Supporting Information to the Manuscript Entitled**

**“Oxidative Carboxylation of 1-Decene to 1,2-Decylene Carbonate”**

Rebecca V. Engel, Raiedhah Alsaiari, Ewa Nowicka, Samuel Pattisson, Peter J. Miedziak, Simon A. Kondrat, David J. Morgan, and Graham J. Hutchings*

Cardiff Catalysis Institute, School of Chemistry, Cardiff University, Main Building, Park Place, Cardiff, CF10 3AT, UK

*Corresponding author: [hutch@cardiff.ac.uk](mailto:hutch@cardiff.ac.uk)


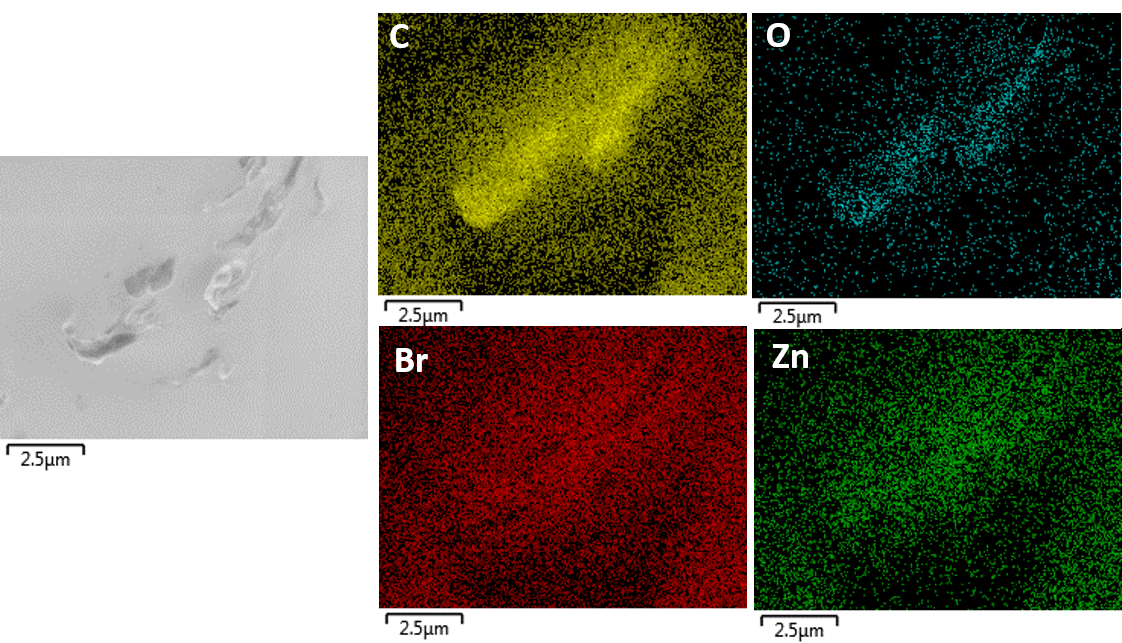


**Figure S1** BSE micrograph and EDX mapping of the GO catalyst used in the epoxidation together with Bu_4_NBr + ZnBr_2_


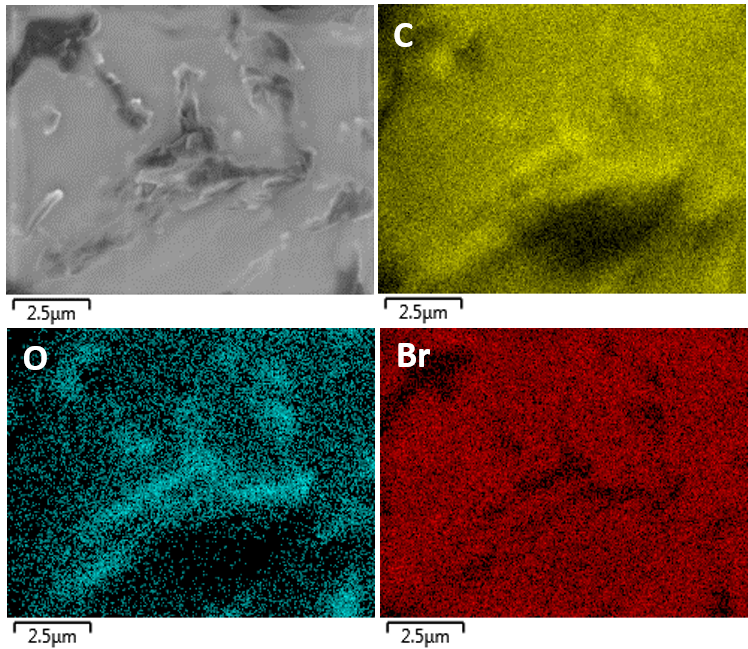


**Figure S2** BSE micrograph and EDX mapping of the GO catalyst used in the epoxidation together with Bu_4_NBr


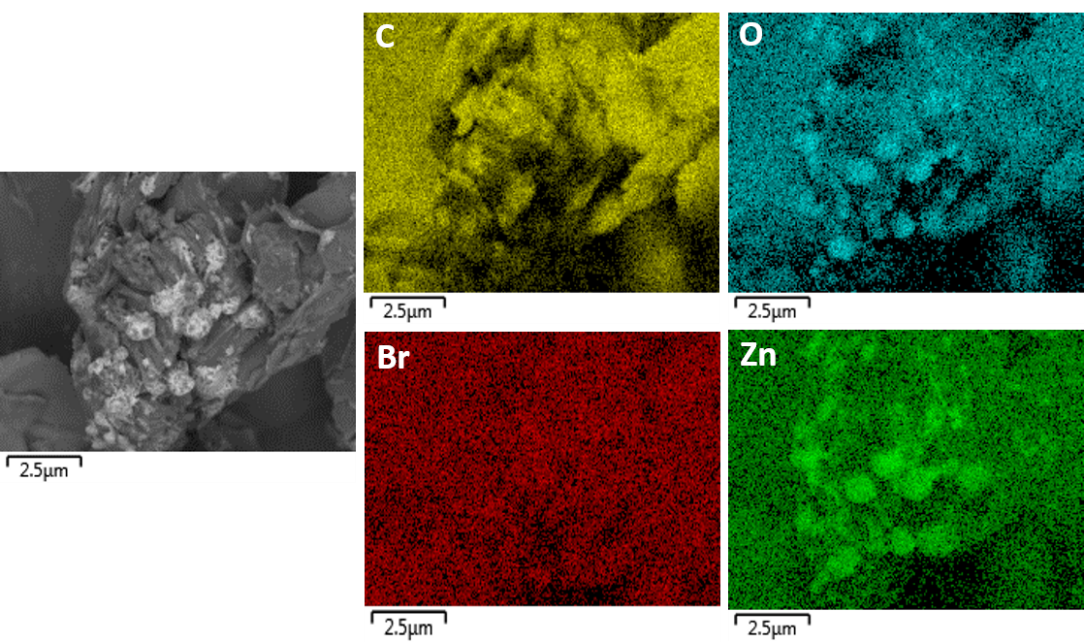


**Figure S3** BSE micrograph and EDX mapping of the GO catalyst used in the epoxidation together with ZnBr_2_


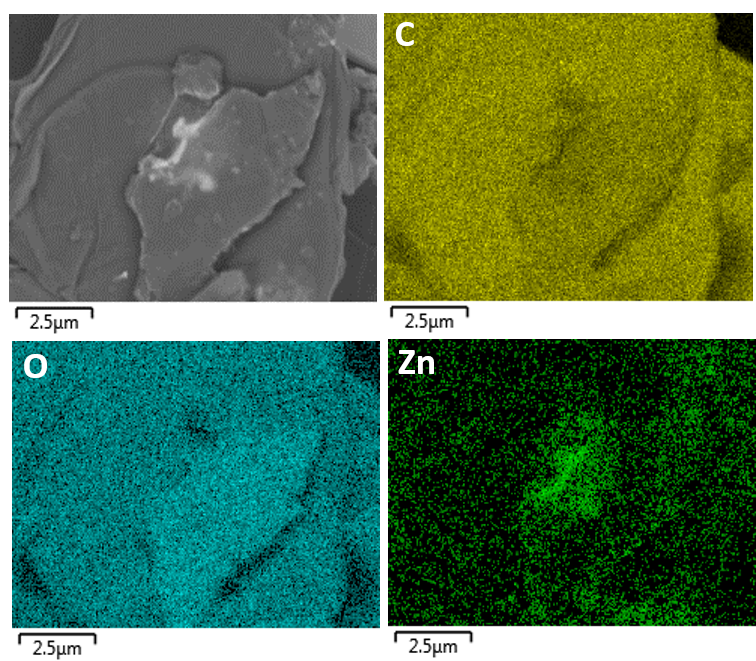


**Figure S4** BSE micrograph and EDX mapping of the GO catalyst used in the epoxidation together with 10x less Bu_4_NBr + ZnBr_2_


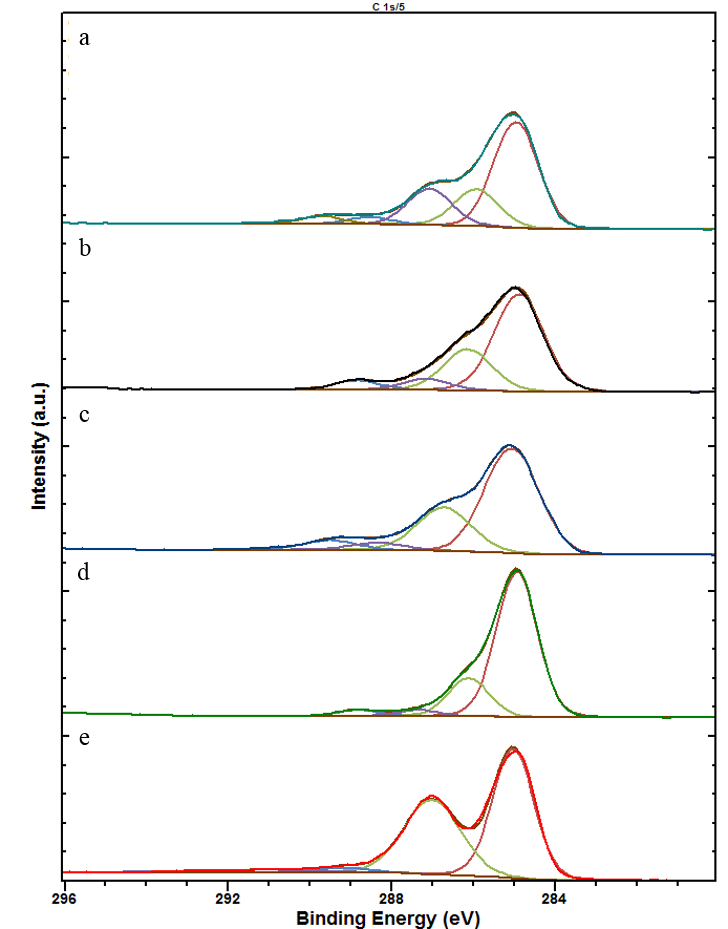


**Figure S5** XPS analysis (C 1s) of the spent (a-d) and fresh (e) GO catalysts; a) ZnBr_2_ b) Bu_4_NBr c) 10x less Bu_4_NBr + ZnBr_2_ d) standard amounts Bu_4_NBr + ZnBr_2_
